# Validation of a Multiparametric, Automated, Macroarray‐Based Assay for the Detection of Autoantibodies in Autoimmune Liver Disease

**DOI:** 10.1002/jcla.70213

**Published:** 2026-04-08

**Authors:** Luigi Raganelli, Tommaso Bandini, Matilde Ilacqua, Helena Cerutti, Alessandra Cartocci, Giulia Tesi, Andrea Ianniello, Thomas Massari, Mirko Berretti, Roberto Centini, Michele Norelli, Alessandra Brogi, Giampaola Pesce, Danilo Villalta, Brunetta Porcelli

**Affiliations:** ^1^ DIESSE Diagnostica Senese S.P.A. Società Benefit Siena Italy; ^2^ Dept of Biotechnology, Chemistry and Pharmacy University of Siena Italy; ^3^ Dept of Medicine, Surgery and Neuroscience University of Siena Siena Italy; ^4^ Dept of Internal Medicine (DIMI) University of Genova Genova Italy; ^5^ Autoimmunology Diagnostic Lab IRCCS Ospedale Policlinico San Martino Genova Italy; ^6^ Immunology and Allergology Lab, Azienda Ospedaliera Friuli Occidentale S. Maria Degli Angeli Hospital Pordenone Italy; ^7^ Dept Medical Biotechnology University of Siena, UOC Clinical Pathology Lab, S. Maria Alle Scotte Hospital, AOU Senese Siena Italy

**Keywords:** autoimmune hepatitis, ELISA, linearity, multiparametric, primary biliary cholangitis, sensitivity, specificity

## Abstract

**Background:**

The Presence of autoantibodies plays a central role in Autoimmune Liver Diseases (AiLD) diagnosis and classification: such antibodies discriminate between distinct subtypes of the AiLD, including Primary Biliary Cholangitis (PBC) and Autoimmune Hepatitis (AIH), and facilitate diagnosis of the overlap syndromes. This paper describes the optimization and validation of a novel procedure to quantify autoantibodies using a multiparametric test and a fully automated device.

**Methods:**

The CHORUS Macroarray Liver Diseases kit was optimized to detect the following biomarkers: autoantibodies anti‐AMA‐M2, anti‐Sp100, and anti‐gp210 for PBC and autoantibodies anti‐LKM‐1, anti‐LC1, and anti‐SLA/LP for AIH types 2 and 3. To validate the assay, the limit of detection, the limit of quantitation (LoQ), precision, specificity, and sensitivity were determined. The assay was also compared with a predicate ELISA that singularly determined each antigen.

**Results:**

A total of 216 serum samples were used to validate the assay, including 91 samples from patients with AIH or PBC and 125 samples from age‐matched blood donors serving as controls. The assay was linear in the range of concentrations between the LoQs determined for each autoantibody, accurate, and precise. When compared with the predicate ELISA, the assay was sensitive and specific, with excellent agreement for each autoantibody. PBC and AIH markers were correlated, and no interaction was present between the two groups.

**Conclusion:**

The CHORUS Macroarray Liver Diseases assay was accurate, sensitive, and specific for the detection of autoantibodies in AiLD and may become a useful tool for rapidly obtaining multiparametric information using a minimal sample volume.

## Graphical abstract

The assay exploited a multiparametric and fully automated device applied to CHORUS EVO to validate that the CHORUS Macroarray Liver Diseases kit is a novel procedure for detecting autoantibodies of AIH and PBC. The assay resulted as linear, accurate, sensitive, specific, and able to distinguish between PBC and AIH.

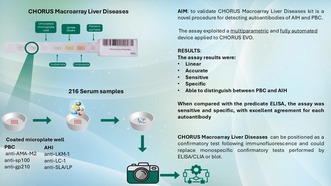

## 1 Introduction

Autoimmune liver diseases (AiLD) represent a broad range of disorders that affect hepatocytes in autoimmune hepatitis (AIH), and cholangiocytes in primary biliary cholangitis (PBC) and primary sclerosing cholangitis (PSC) [1, 2]. The presence of autoantibodies plays a central role in the AiLD diagnosis and classification: such antibodies discriminate between distinct subtypes of the AiLD and facilitate diagnosis of the overlapping syndromes [1, 2, 3, 4].

AIH is a non‐resolving chronic liver disease that affects mainly women and is characterized by hypergammaglobulinemia even in the absence of cirrhosis and circulating autoantibodies association with human leukocyte antigens DR3 or DR4, interface hepatitis on liver histology, and a favourable response to immunosuppression [5]. Its prevalence ranges from 16 to 18 per 100,000 inhabitants in Europe, and it is increasing in both women and men; the disease can affect patients at any age [6].

According to the pattern of autoantibodies detected, a subclassification has been proposed. AIH‐1 accounts for almost 90% of AIH cases, with an onset at any age and variable histopathological severity; it is characterized by the presence of anti‐nuclear antibodies (ANA) and/or anti‐smooth muscle antibodies (SMA), and/or antibodies against soluble liver antigens/live pancreas (anti‐SLA/LP). AIH‐2 accounts for up to 10% of AIH cases, with an onset especially during childhood and young adulthood, and presents anti‐liver cytosol‐1 (anti‐LC‐1) and liver kidney microsomal antibodies (anti‐LKM). These latter are specifically directed against cytochrome P4502D6, a cytoplasmic protein with a molecular weight of approximately 50 kDa, localized in hepatocytes and proximal renal tubules.

PBC is a chronic inflammatory autoimmune cholestatic liver disease, which culminates in end‐stage biliary cirrhosis if untreated. In European populations, the estimated incidence is between 1–2 per 100,000 population per year, with a prevalence of 1.9–40.2 per 100,000 [7, 8, 9].

The diagnostic criteria for PBC are: (i) a persistent elevation of ALP (for more than six months) (ii) a liver biopsy showing histological evidence of chronic cholangitis of the interlobular bile ducts (iii) and the presence of AMA at a concentration greater than 1:40, which is considered a highly predictive criterion for the disease. The simultaneous presence of all three criteria allows for a definitive diagnosis of PBC, whereas having only two criteria makes the diagnosis probable but not certain. Numerous studies have shown that AMA, which is found in approximately 95% of cases, can appear during the asymptomatic phase of the disease, even several years before biochemical indicators of liver damage emerge. It is now established that AMA levels do not correlate with either disease severity or progression, making their monitoring during clinical follow‐up unnecessary. It is estimated that around 5% of PBC patients are AMA‐negative, a condition that may lead to delayed or missed diagnosis. To overcome this diagnostic limitation and improve the sensitivity of serological testing, additional autoantibody specificities have been identified and associated with PBC: anti‐Sp100, which targets a 53‐kDa protein localized in promyelocytic leukemia nuclear bodies, and anti‐gp210, which targets a nuclear pore glycoprotein. These markers are used in the diagnosis of PBC in AMA‐negative patients and are included in the current diagnostic criteria for PBC.

Autoantibodies are the hallmark of AiLD, and their detection represents an important part of the diagnostic workup. Indirect immunofluorescence is the main technique used for routine testing for autoantibodies ANA, SMA, LKM‐1, LC1, and AMA; it should be performed on freshly frozen rodent substrate that usually includes the kidney, liver, and stomach [6]. In recent years, line immunoassays and enzyme‐linked immunosorbent assays (ELISA), using recombinant proteins which improve test sensitivity and specificity, have been developed to detect AiLD autoantibodies and confirm the immunofluorescence positivity [10, 11]. For anti‐SLA/LP antibodies, ELISA and line immunoassays are the only diagnostic tests, whose exact target antigens have been identified on a molecular level and are used in solid phase assays [6, 12, 13, 14, 15].

In AIH, autoantibody titres and specificity may vary during the disease, and seronegative individuals at diagnosis may express the conventional autoantibodies later in the disease course [6, 16]. In fact, repeated testing may allow autoantibody detection and, thus, correct disease diagnosis and classification [1, 6, 16]. In adults, autoantibody titres correlate only roughly with disease activity, clinical course, and treatment response [5], and therefore, they do not need to be monitored regularly unless a significant change in the clinical phenotype does appear.

In PBC, immunofluorescence staining on HEp‐2 substrates showing nuclear dots (suggestive of anti‐Sp100 reactivity) and/or perinuclear rim patterns (suggestive of anti‐gp210 reactivity) is diagnostically useful in approximately 5%–10% of patients who are AMA‐negative, depending on the assay used [13, 14].

The detection of specific autoantibodies is, therefore, required to achieve a differential diagnosis between AIH and PBC and among other liver diseases [6].

In this paper, a novel procedure for detecting autoantibodies of AIH and PBC, the CHORUS Macroarray Liver Diseases kit, was described. The assay exploited a multiparametric and fully automated device applied to CHORUS EVO, a new instrument from DIESSE Diagnostica Senese S.p.A. Società Benefit, and was validated in terms of accuracy, efficiency, and reproducibility, according to guidelines and by comparison with other commercially available tests. As the availability of an automated multiparametric assay that can simultaneously identify specific autoantibody patterns may be important for prompt and highly reliable differential diagnosis, CHORUS Macroarray Liver Diseases kit was also tested with serum samples from patients to evaluate its sensitivity and specificity.

## 2 Methods

### 2.1 Antigens

CHORUS Macroarray Liver Diseases kit (DIESSE Diagnostica Senese S.p.A. Società Benefit, Monteriggioni (SI), Italy CE‐marked) was optimized to detect the following biomarkers: autoantibodies anti‐AMA‐M2, anti‐Sp100, and anti‐gp210 for PBC and autoantibodies anti‐LKM‐1, anti‐LC‐1, and anti‐SLA/LP for AIH.

All antigens were full recombinant constructs of human proteins expressed in eukaryotic systems, except for SLA/LP, which was produced in bacterial cells. M2 consisted of a triple hybrid recombinant molecule (r‐MIT3) that contained the autoepitopes of pyruvate dehydrogenase (PDC‐E2), branched‐chain 2‐oxoacid dehydrogenase complex (BCOADC‐E2), and 2‐oxoglutarate dehydrogenase complex (OGDC‐E2) in an equal‐mass mixture.

### 2.2 CHORUS Macroarray Liver Diseases Assay Protocol

The CHORUS Macroarray Liver Diseases is based on a modified indirect ELISA method [17]. The main difference with a traditional indirect ELISA assay is that many antigens were dispensed in the same well using a predetermined layout, small working volumes were requested, thus limiting reagent consumption, and a precipitant reagent discriminated positive from negative spots.

Briefly, each well of the CHORUS Macroarray Liver Diseases kit is coated with the relevant protein antigens using a non‐contact printer, which can create a matrix of multiple biomaterials on a functionalized surface for multiparametric analysis. Reference spots within the matrix allow the well to be read in any orientation and serve as negative controls if a specific antigen signal is absent. All visible spots are automatically identified based on their predefined layout. During the assay, the immunoglobulins in the serum sample bind to specific antigens coated on the plate, forming antigen–antibody complexes. Excess antibodies are washed away, and a secondary antibody labelled with horseradish peroxidase (HRP) is added to detect the complexes. After another wash, the bound HRP reacts with a 3,3′,5,5′‐tetramethylbenzidine substrate, producing a permanent precipitate on each reactive micro spot [18].

During the study, the entire procedure was accomplished using the CHORUS EVO instrument, a fully automated multiparametric diagnostic system developed by DIESSE Diagnostica Senese SpA Società Benefit. This analyser is specifically designed for immunometric assays with serum samples and ready‐to‐use single test devices. It features three reading systems: photometric for ELISA tests, chemiluminescence for immunoassays, and a camera‐based analyser for macroarray tests. The camera captures images of the entire well, and the onboard algorithm identifies regions of interest (ROI) based on morphological and positional criteria. Each ROI generates a raw pixel readout, which is then analysed both qualitatively and quantitatively.

### 2.3 Study Design

This was an inter‐laboratory validation study as serum samples were obtained from the serum bank of three Italian hospitals. All samples were fully anonymized prior to analysis; therefore, the study did not require informed consent. The study was conducted according to the ethical principles reported in the Declaration of Helsinki in its latest revision. Data collection and evaluation were conducted without access to information regarding sampling time, patient prognosis, or ongoing treatments. Detailed clinical and demographic characteristics, apart from age, could not be reported, as the participating centres supplied anonymized serum samples labelled solely with the essential information described above and without any additional clinical data. The only available information was that the sera had been collected from patients undergoing testing for diagnostic markers of autoimmune liver disease.

### 2.4 Assay Validation

The Limit of Detection (LoD) and Limit of Quantitation (LoQ) were determined in accordance with the 2nd Edition of the Clinical & Laboratory Standards Institute's “Evaluation of Detection Capability for Clinical Laboratory Measurement Procedures” [19]. For this determination, two kit lots were used and detected by two instruments.

The Limit of Detection (LoD) was estimated by a parametric analysis where the LoD was set as the maximal value obtained for each reagent lot, and the pixel count was then converted into Arbitrary Unit (AU)/mL. The Limit of Quantitation (LoQ) was determined in serum samples, which were run in triplicate in a CHORUS EVO instrument on three different days using two different lots; references at known concentrations were tested for each autoantibody. The test was optimized to avoid cross‐reactivity with the tested parameters and interferences with hemoglobin, bilirubin, triglycerides, and rheumatoid factor.

To determine the linearity, two samples were assayed in duplicate, and the results were plotted on an XY graph where the horizontal axis represented the expected values and the vertical axis the results; to verify the presence of non‐linearity, a test for linear and polynomial coefficients was carried out [20].

Precision and repeatability were assayed using three samples (four for anti‐AMA‐M2) at increasing concentrations to ensure the analysis across the entire dosage range. For the intra‐assay precision, the analysis was performed with one lot on one CHORUS EVO instrument by one operator. Each analyte concentration was tested in six replicates. For the inter‐assay precision, each analyte concentration was tested with one lot on one CHORUS EVO instrument over six runs. The inter‐lot precision was evaluated with three lots on one CHORUS EVO instrument by one operator; each sample was tested over six runs. The inter‐instrument precision was analyzed with one lot on three CHORUS EVO instruments by one operator, and each sample was tested over six runs with the three instruments. In all experiments, one control sample was added to judge the acceptability of the run. A coefficient of variation (CV) ≤ 20% was considered acceptable.

Lastly, to determine specificity and sensitivity, the CHORUS Macroarray Liver Diseases kit was compared with ELISA that singularly quantified each antigen [QUANTA Lite AMA‐M2 ELISA, Ref. 704,540; QUANTA Lite Sp100 ELISA, Ref. 708,990; QUANTA Lite gp210 ELISA, Ref. 708,995; QUANTA Lite LKM‐1 ELISA, Ref. 708,745; QUANTA Lite SLA ELISA, Ref. 708,775 from Inova Diagnostics; EUROIMMUN Anti‐LC‐1 ELISA (IgG), Ref. EA 1307–9601 G from Euroimmun Italia]. All the assays were performed following the manufacturer's instructions. The six ELISA tests used for comparison were already validated assays, and the manufacturing companies were well‐known in routine diagnostics for autoimmune diseases; therefore, they were considered reliable.

### 2.5 Statistical Analysis

Qualitative variables were summarized with absolute frequencies and percentages. To compare the CHORUS Macroarray Liver Diseases with single antigen ELISA, the Cohen's k coefficient was calculated; the Cohen's k coefficient indicated a poor agreement if it ranged from 0.0 to 0.4; fair agreement between 0.4–0.6, good agreement between 0.6–0.8, and excellent agreement between 0.8–1.0. The cut‐off was determined using data of positive, negative, and equivocal sera. The criteria for defining the acceptability performance were established based on the current state of the art of multiplex and singleplex assays commercially available on the market: SeraSpot HepAk‐7 IgG by Seramun; EUROLINE Blot (AMA M2, M2‐3E, Sp100, PML, gp210, LC‐1, LKM‐1, SLA/LP, Ro‐52) (EUROIMMUN); QUANTA Lite SLA ELISA (INOVA Diagnostics, Werfen); QUANTA Lite M2 EP (MIT3) ELISA (INOVA Diagnostics, Werfen); QUANTA Lite Sp100 ELISA (INOVA Diagnostics, Werfen); QUANTA Lite gp210 ELISA (INOVA Diagnostics, Werfen); QUANTA Lite LKM‐1 ELISA (INOVA Diagnostics, Werfen); Anti LC‐1 ELISA (IgG) (EUROIMMUN). Results were analysed applying a Receiver‐Operating Characteristic (ROC) plot generated with the software Analyse‐it; the sensitivity and specificity > 85% and negative and positive predictive value > 80% were accepted. The value that maximized all the sensitivity, specificity, positive, and negative predicted values was considered the final cut‐off. Spearman's correlation coefficients were also determined to evaluate the correlation between markers, and a false discovery rate (FDR) correction was applied. A *p*‐value < 0.05 was considered statistically significant. All analyses were performed using Analyse‐it Software version 5.96 (Analyse‐it Software Ltd., United Kingdom).

## 3 Results

A total of 216 serum samples were used to validate the CHORUS Macroarray Liver Diseases kit: 91 were obtained from patients with suspected AIH (34,1%) and PBC (65,9%), and 125 were from blood donors matched for age with the autoimmune liver disease cohort. Among patients with AIH, 6.5% were in the paediatric age group, 87% in the adult age group, and 6.5% were aged > 70 years. Among patients with PBC, 65% were in the adult age group, and 35% were aged > 70 years.

The samples from blood donors were used to determine the limits of quantification for all autoantibodies, and Table 1 summarizes the results. Based on values of the LoD and ULoD, the dosing ranges were as follows: AMA‐M2 5–65.0 AU/mL, Sp100 4.0–70.0 AU/mL, gp210 7.0–80.0 AU/mL, LKM‐1 3.0–60.0 AU/mL, LC‐1 3.0–70.0 AU/mL, SLA/LP 3.0–65.0 AU/mL. The dosing ranges included both negative and positive values that define the pathological condition. The reference interval of the normal range is reported in the last row of Table 1.

**TABLE 1:** Limits of quantification. For each autoantibody, the LoD and the ULoQ and LLoQ were determined. Clinical cut‐off with negativity, positivity and the normal range were also reported. The ‘Normal range’ represents the 95th percentile (or mean ± 2SD) derived from healthy donor samples. The ‘Negative/Positive value’ is the clinical decision threshold based on the ROC‐optimized cut‐off.

	AMA‐M2	Sp100	gp210	LKM‐1	LC‐1	SLA/LP
	AU/ml	AU/ml	AU/ml	AU/ml	AU/ml	AU/ml
Blank	1.0	1.0	1.0	1.0	1.0	1.0
LoD	5.0	4.0	7.0	3.0	3.0	3.0
LLoQ	12.7	13.8	14.9	11.4	13.8	16.1
ULoQ	65.6	70.8	80.1	60.4	70.2	65
Dosing Range	5–65.0	4.0–70.0	7.0–80.0	3.0–60.0	3.0–70.0	3.0–65.0
Clinical Cut‐off	15.0	19.0	20.0	19.0	18.0	22.0
Negative value	< 13.5	< 17.0	< 18.0	< 17.0	< 16.0	< 20.0
Positive value	> 16.5	> 21.0	> 22.0	> 21.0	> 20.0	> 24.0
Normal range	< 5.0	< 4.0	< 7.0	< 3.0	< 3.0	< 3.0

**FIGURE 1:**
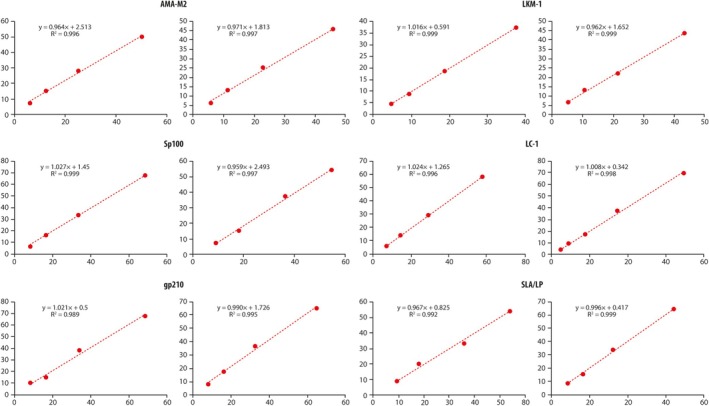
Linearity. A linearity between the expected concentration and the results was found after testing two samples in a predetermined range of values.

The linearity was also tested, and for each parameter, an R^2^ > 0.99 was obtained, with non‐significant coefficients for x^2^ and x^3^ (Figure 1). The mean recovery percentage (difference between expected and actual values) was within ±15% for each sample and parameter analysed.

Table 2 reports the results of precision tests. When the samples were evaluated between batches and within the same run, the coefficient of variation varied from 1.3% to 18.8%, thus indicating that the assay was reliable and accurate. The analytical analyses with interfering substances indicated that haemoglobin, bilirubin, triglycerides, and Rheumatoid Factor did not affect the assay results (Tables S1–S4). Cross‐reactivity data were negative, thus suggesting that no aspecific cross‐reactions occurred (Table S5A,B).

**TABLE 2:** Precision evaluation. Precision was evaluated within the run, between runs, between batches, and between instruments.

Parameter	Sample	Within‐run precision		Between‐run precision	
		Value (AU/ml)	CV%	Value (AU/ml)	CV%
AMA‐M2	1	62.0	1.3	56.3	10.2
	2	48.6	9.3	50.6	13.4
	3	37.6	6.6	41.8	8.9
	4	25.6	18.6	21.9	18.5
Sp100	1	56.1	10.7	51.6	11.8
	2	43.5	7.5	42.0	11.8
	3	23.6	10.7	27.1	10.7
gp210	1	67.4	10.7	60.0	16.0
	2	47.3	15.9	55.8	16.4
	3	26.6	14.9	28.5	15.5
LKM‐1	1	48.8	13.6	45.0	4.2
	2	39.7	9.2	39.6	10.7
	3	20.3	13.7	22.8	14.6
LC‐1	1	54.7	3.5	57.3	12.9
	2	35.2	9.7	42.4	17.4
	3	28.8	10.1	24.1	16.2
SLA/LP	1	50.8	17.7	51.1	13.4
	2	45.2	14.9	47.7	18.4
	3	33.3	10.3	32.8	12.1

		Precision between batches		Precision between instruments	
Parameter	Sample	Value (AU/ml)	CV%	Value (AU/ml)	CV%
AMA‐M2	1	57.2	10.4	60.5	4.1
	2	50.1	7.2	54.8	11.3
	3	41.5	2.1	42.2	9.2
	4	26.5	14.6	25.5	14.1
Sp100	1	42.0	13.2	55.2	17.2
	2	37.9	9.7	47.3	16.7
	3	28.1	13.0	26.8	18.2
gp210	1	57.1	15.7	63.5	14.7
	2	52.0	11.0	53.3	17.9
	3	26.0	9.2	27.0	16.3
LKM‐1	1	42.5	16.1	47.0	15.9
	2	39.8	6.9	52.0	11.4
	3	22.1	11.7	21.6	10.2
LC‐1	1	54.6	11.1	57.9	10.1
	2	37.7	5.4	39.0	10.6
	3	26.1	9.8	28.6	13.8
SLA/LP	1	51.6	7.3	50.4	15.1
	2	43.9	11.7	50.2	15.8
	3	32.6	18.8	33.8	12.0

**FIGURE 2:**
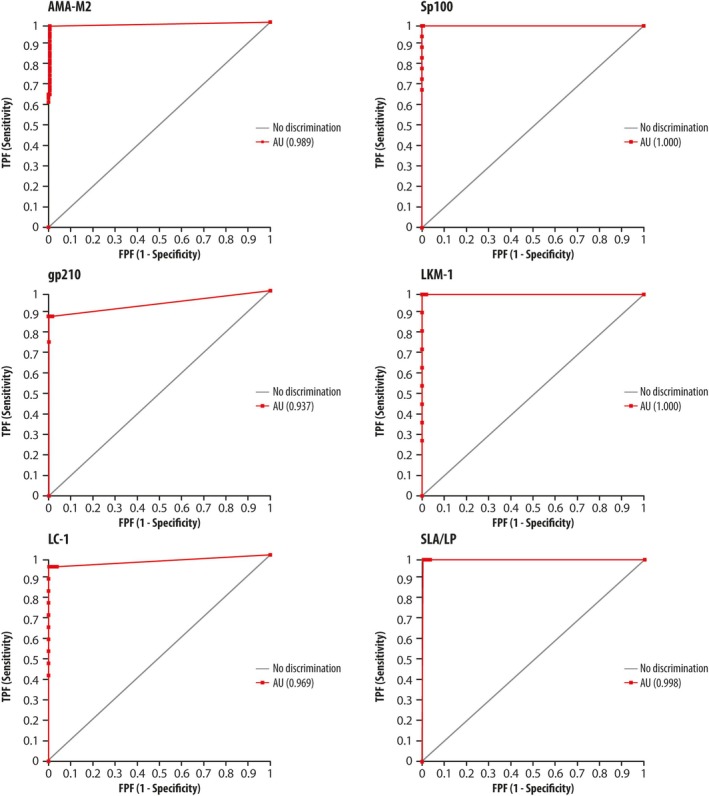
Receiver Operating Curve plots. The data are plotted on a graph showing: On X axis the False Positive rate (1‐specificity) – the probability of incorrectly diagnosing a case as positive when its true state is negative. On Y axis the True Positive Rate (sensitivity) – the probability of correctly diagnosing a positive case across all decision levels for the diagnostic test.

The sensitivity and specificity of the assay were assessed by comparing the CHORUS Macroarray Liver Disease kit with the predicate devices QUANTA‐Lite series and EUROIMMUN Anti‐LC‐1 ELISA (IgG). The Cohen's k coefficient was determined for each comparison and, in all cases, the agreement was excellent, as the coefficient ranged from 0.90 to 1.00 (Table 3). The cut‐offs calculated for each parameter were as follows: AMA‐M2: 15.0 AU/mL, Sp100: 19.0 AU/mL, gp210: 20.0 AU/mL, LKM‐1: 19.0 AU/mL, LC‐1: 18.0 AU/mL, SLA/LP: 22.0 AU/mL. These values were confirmed by ROC plots (Figure 2).

**TABLE 3:** Sensitivity and specificity analysis. The comparison between CHORUS Macroarray Liver Disease and the predicate ELISA for each autoantibody indicated high sensitivity and specificity. For all autoantibodies, the agreement was excellent, as demonstrated by the Cohen's k.

Parameter	AMA‐M2	Sp100	gp210	LKM‐1	LC‐1	SLA/LP
Predicate	QUANTA Lite AMA‐M2 ELISA	QUANTA Lite Sp100 ELISA	QUANTA Lite gp210 ELISA	QUANTA Lite LKM‐1 ELISA	EUROIMMUN Anti‐LC‐1 ELISA (IgG)	QUANTA Lite SLA ELISA
Accuracy %	98.6	100.0	99.5	99.5	99.1	99.1
Sensitivity	96.3	100.0	87.5	100.0	94.1	100.0
CI 95%	87.4–99.0	83.2–99.8	52–97.5	72.2–99.6	72.9–98.8	67.5–99.6
Specificity	99.4	100.0	100.0	99.5	99.5	99.0
CI 95%	96.6–99.9	98.1–100	98.2–100.0	97.3–99.9	97.2–99.9	96.6–99.7
Cohen's k	0.96	1.00	0.93	0.95	0.93	0.90

The correlation study was conducted using patient samples. Spearman's analysis revealed two distinct patterns of autoantibodies: one group included anti‐AMA‐M2, anti‐Sp100, and anti‐gp210; the other comprised anti‐LKM‐1, anti‐LC‐1, and anti‐SLA/LP. No correlation was found between these two groups, confirming the test's strong performance (Table 4). Moderate correlations were observed within each group: between anti‐AMA‐M2 and anti‐Sp100, anti‐AMA‐M2 and anti‐gp210, and among anti‐LKM‐1, anti‐LC‐1, and anti‐SLA/LP.

**TABLE 4:** Spearman's correlation between markers. In bold, the significant correlation according to Spearman (*p* < 0.05).

Marker 1	Marker 2	Spearman's correlation	*p* adjusted
AMA‐M2	Sp100	**0.481**	**< 0.001**
AMA‐M2	gp210	**0.354**	**< 0.001**
AMA‐M2	LKM‐1	−0.041	0.688
AMA‐M2	LC‐1	−0.109	0.223
AMA‐M2	SLA/LP	−0.107	0.223
Sp100	gp210	0.151	0.068
Sp100	LKM‐1	−0.085	0.357
Sp100	LC‐1	−0.014	0.85
Sp100	SLA/LP	0.027	0.801
gp210	LKM‐1	−0.060	0.515
gp210	LC‐1	−0.078	0.381
gp210	SLA/LP	0.013	0.85
LKM‐1	LC‐1	**0.540**	**< 0.001**
LKM‐1	SLA/LP	**0.306**	**< 0.001**
LC‐1	SLA/LP	**0.517**	**< 0.001**

## 4 Discussion

This work describes the validation of the CHORUS Macroarray Liver Disease kit, a multiparametric and fully automated device for the simultaneous determination of markers of PBC and AIH in serum samples. The results indicate that the assay was accurate and reliable in quantifying these markers and able to distinguish between PBC and AIH. The calibration interval established by the lower and upper LoQ covered the entire range of the protein, thus guaranteeing that CHORUS EVO could accurately determine the concentration of serum markers and correctly discriminate physiological from pathological conditions. The assay was also linear within the calibration range and specific for each antigen. When compared with single antigen ELISA, the CHORUS Macroarray Liver Disease kit showed excellent agreement, thus confirming that the multiparametric assay maintained the necessary sensitivity and specificity. The correlation between Sp100 and gp210 was not statistically significant in our study, most likely because the prevalence of both markers was very low [15]. This can be explained by the fact that the sample used for the test validation was random, as no selection of the samples to be analysed was performed. The lack of overlap was because the samples were selected for antibody positivity for the validation of the test; therefore, no clinical consideration regarding prevalence and/or co‐positivity can be made.

The experiences with other multiparametric assays have been reported in the literature. A line immunoassay attempted to measure nine autoantibodies (AMA‐M2, M2‐3E, Sp100, PML, gp210, Ro‐52, LKM‐1, LC‐1, and SLA/LP) simultaneously in patients with PBC, AIH, and PBC‐AIH overlap; the assay correctly identified the differential diagnosis, and only a few patients tested positive for 2 or more autoantibodies [11]. The multiplexed line‐blot Autoimmune Liver Disease Profile 2 (ALD2, Euroimmun) was developed to detect several autoantibodies: anti‐LKM1, anti‐LC1, anti‐SLA/LP, anti‐AMA‐M2, anti‐gp210, anti‐Sp100, and anti‐PML. This assay demonstrated higher sensitivity than immunofluorescence for detecting anti‐LC1. Results from a cohort study of paediatric and adult samples confirmed that combining this analytical method with immunofluorescence was especially useful when initial results were inconclusive [10]. Another multiplex line/dot blot (D‐Tek) was tested to detect autoantibodies from serum samples of the Dutch EQC program. For anti‐AMA, a concordance rate of 97% was observed [16].

Our results indicated that the CHORUS Macroarray Liver Disease can discriminate between AIH and PBC, and the presence of the markers for each disease can confirm the diagnosis after immunofluorescence.

Simultaneous screening based on a protein array system and ELISA is a consolidated methodology to detect and quantify a variety of proteins from serum samples [21, 22, 23]. The CHORUS Macroarray Liver Disease kit takes advantage of some important properties of miniaturized ligand‐binding assays [24, 25, 26]: (i) the sensitivity increases because the binding reaction occurs at the highest possible target concentration and the capture‐molecule–target complex is found only in the small area of the microspot, thus resulting in a high signal; (ii) the analytic conditions are independent of the actual volume of sample used, thus providing results with low sample consumption; (iii) the possibility to include in the array positive and negative control spots and/or internal calibration spots leads to robust and reliable diagnostic assays.

Since the diagnosis of autoimmune liver disease is typically performed using immunofluorescence or immunoblot, with other assays serving as confirmation, the ability to quantify six autoantibodies from a small serum sample is particularly useful in clinical practice, where sample availability is often a constraint. Furthermore, the macroarray can be used as a first‐level screening test in laboratories that do not perform immunofluorescence, or as a second‐level test to confirm via more specific assays (M2 and LKM), to detect positive markers (gp210/Sp100 or SLA), or to analyse markers not detectable by immunofluorescence (SLA).

The CHORUS Macroarray Liver Disease kit is an automated test that can reduce the variability of quantification and contribute to overcoming and/or limiting potential mistakes and misdiagnoses associated with human errors, even if the immunoassay readout is automated.

As major limitations, it should be noted that this study was exclusively aimed at presenting the validation procedure of the CHORUS Macroarray Liver Diseases kit. It was not possible to provide details on clinical and demographic characteristics because the participating centres provided anonymized serum samples annotated only with the required information reported above, without any additional clinical data. The experimental activity was, therefore, limited to measuring autoantibodies in the serum samples, independently of the clinical or therapeutic context.

The new multiplex assay was compared with the individual tests currently used in routine hospital laboratories to assess concordance and demonstrate its reliability. This approach allows us to validate our test and support its introduction into routine laboratory practice, and further prospective data on its diagnostic use are warranted to better describe its clinical accuracy, diagnostic performance, and feasibility. The demonstrated analytical equivalence to established, single‐parameter confirmatory ELISA assays suggests that the CHORUS Macroarray could potentially serve as an efficient, consolidated confirmatory test, potentially replacing multiple single‐plex assays. This proposed clinical utility should be validated in prospective studies.

## 5 Conclusion

The results described in this study indicate that CHORUS Macroarray Liver Diseases was accurate and reliable in performing a multiparametric analysis of autoantibodies detected in autoimmune liver diseases. The test is completely automated and requires a minimal amount of serum samples to determine the presence of six autoantibodies simultaneously. It may become a useful tool in clinical practice.

## Funding

Medical writing services and article processing charges are funded by DIESSE Diagnostica Senese S.p.A. Società Benefit.

## Ethics Statement

The study was conducted according to the ethical principles reported in the Declaration of Helsinki in its latest revision.

## Consent

As all samples had been anonymized after the discard, this study did not require informed consent.

## Conflicts of Interest

Luigi Raganelli, Tommaso Bandini, Helena Cerutti, Giulia Tesi, Andrea Ianniello, Thomas Massari, Mirko Berretti, Roberto Centini, Michele Norelli, Alessandra Brogi, declare to be “DIESSE Diagnostica Senese S.p.A. Società Benefit” employees. No other conflicts of interest has been reported.

## Supporting information


**Table S1:** Analytical analysis in presence of increasing concentration of haemoglobin.
**Supplementary Table 2** Analytical analysis in presence of increasing concentration of bilirubin.
**Supplementary Table 3** Analytical analysis in presence of increasing concentration of triglyceride.
**Supplementary Table 4** Analytical analysis in presence of increasing concentration of Rheumatoid factor.
**Supplementary Table 5A** Cross‐reactivity data for AMA, Sp100, and gp210.
**Supplementary Table 5B** Cross‐reactivity data for LKM‐1, LC‐1, and SLA/LP.

## Data Availability

The data that support the findings of this study are available from the corresponding author upon reasonable request.
