# Supplementary material for: Validation of a Multiparametric, Automated, Macroarray‐Based Assay for the Detection of Autoantibodies in Autoimmune Liver Disease
Source: J Clin Lab Anal. 2026 Apr 8;40(11):e70213. doi: 10.1002/jcla.70213 (PMC13267151; doi:10.1002/jcla.70213)
Supplement: Supplementary file 1 — Table S1: Analytical analysis in presence of increasing concentration of haemoglobin. Supplementary Table 2 Analytical analysis in presence of increasing concentration of bilirubin. Supplementary Table 3 Analytical analysis in presence of increasing concentration of triglyceride. Supplementary Table 4 Analytical analysis in presence of increasing concentration of Rheumatoid factor. Supplementary Table 5A Cross‐reactivity data for AMA, Sp100, and gp210. Supplementary Table 5B Cross‐reactivity data for LKM‐1, LC‐1, and SLA/LP. [file JCLA-40-e70213-s001.docx]

**Supplementary Table 1** Analytical analysis in presence of increasing concentration of haemoglobin

|  |  | **Haemoglobin** | | | | | | | | |
| --- | --- | --- | --- | --- | --- | --- | --- | --- | --- | --- |
| **Parameter** | **Sample** | **Level 1 (10 mg/ml)** | | | **Level 2 (5 mg/ml)** | | | **Level 3 (2,5 mg/ml)** | | |
|  |  | **Control AU/mL** | **Spiked AU/mL** | **%diff** | **Control AU/mL** | **Spiked AU/mL** | **%diff** | **Control AU/mL** | **Spiked AU/mL** | **%diff** |
| **AMA** | **Negative Samples** | <5 | <5 | 0 | <5 | <5 | 0 | <5 | <5 | 0 |
|  | **Low Samples** | 25,9 | 28,9 | 12 | 25,9 | 22,3 | 14 | 25,9 | 30,1 | 16 |
|  | **High Samples** | 52,8 | 48,8 | 8 | 52,8 | 62,1 | 18 | 52,8 | 60,3 | 14 |
| **Sp100** | **Negative Samples** | <4 | <4 | 0 | <4 | <4 | 0 | <4 | <4 | 0 |
|  | **Low Samples** | 34,3 | 29,3 | 15 | 34,3 | 33,4 | 3 | 34,3 | 40,1 | 17 |
|  | **High Samples** | 66,2 | 58,2 | 12 | 66,2 | 66,8 | 1 | 66,2 | 62,2 | 6 |
| **gp210** | **Negative Samples** | <7 | <7 | 0 | <7 | <7 | 0 | <7 | <7 | 0 |
|  | **Low Samples** | 28,4 | 24,1 | 15 | 28,4 | 33,9 | 19 | 28,4 | 25,1 | 12 |
|  | **High Samples** | 59,6 | 68,3 | 15 | 59,6 | 70,3 | 18 | 59,6 | 55,4 | 7 |
| **LKM-1** | **Negative Samples** | <3 | <3 | 0 | <3 | <3 | 0 | <3 | <3 | 0 |
|  | **Low Samples** | 36,3 | 29,6 | 18 | 36,3 | 32,7 | 10 | 36,3 | 29,4 | 19 |
|  | **High Samples** | 50,2 | 55,1 | 10 | 50,2 | 59,9 | 19 | 50,2 | 56,3 | 12 |
| **LC-1** | **Negative Samples** | <3 | <3 | 0 | <3 | <3 | 0 | <3 | <3 | 0 |
|  | **Low Samples** | 24,4 | 27,3 | 12 | 24,4 | 20,9 | 14 | 24,4 | 19,9 | 18 |
|  | **High Samples** | 48,5 | 55,2 | 14 | 48,5 | 49,6 | 2 | 48,5 | 54,3 | 12 |
| **SLA/LP** | **Negative Samples** | <8 | <8 | 0 | <8 | <8 | 0 | <8 | <8 | 0 |
|  | **Low Samples** | 32,2 | 27,3 | 15 | 32,2 | 36,6 | 14 | 32,2 | 34,2 | 6 |
|  | **High Samples** | 59,9 | 64,5 | 8 | 59,9 | 50,5 | 16 | 59,9 | 64,6 | 8 |

**Supplementary Table 2** Analytical analysis in presence of increasing concentration of bilirubin

|  |  | **Bilirubin** | | | | | | | | |
| --- | --- | --- | --- | --- | --- | --- | --- | --- | --- | --- |
| **Parameter** | **Sample** | **Level 1 (45 mg/dL)** | | | **Level 2 (18 mg/dL)** | | | **Level 3 (4,5 mg/dL)** | | |
|  |  | **Control AU/mL** | **Spiked AU/mL** | **%diff** | **Control AU/mL** | **Spiked AU/mL** | **%diff** | **Control AU/mL** | **Spiked AU/mL** | **%diff** |
| **AMA** | **Negative Samples** | <5 | <5 | 0 | <5 | <5 | 0 | <5 | <5 | 0 |
|  | **Low Samples** | 25,9 | 22,2 | 14 | 25,9 | 30,1 | 16 | 25,9 | 30,1 | 16 |
|  | **High Samples** | 52,8 | 45,4 | 14 | 52,8 | 55,4 | 5 | 52,8 | 60,3 | 14 |
| **Sp100** | **Negative Samples** | <4 | <4 | 0 | <4 | <4 | 0 | <4 | <4 | 0 |
|  | **Low Samples** | 34,3 | 28,8 | 16 | 34,3 | 30,2 | 12 | 34,3 | 40,1 | 17 |
|  | **High Samples** | 66,2 | 56,3 | 15 | 66,2 | 69,1 | 4 | 66,2 | 63,3 | 4 |
| **gp210** | **Negative Samples** | <7 | <7 | 0 | <7 | <7 | 0 | <7 | <7 | 0 |
|  | **Low Samples** | 28,4 | 25,6 | 10 | 28,4 | 28,8 | 1 | 28,4 | 25,1 | 12 |
|  | **High Samples** | 59,6 | 66,2 | 11 | 59,6 | 66,3 | 11 | 59,6 | 55,4 | 7 |
| **LKM-1** | **Negative Samples** | <3 | <3 | 0 | <3 | <3 | 0 | <3 | <3 | 0 |
|  | **Low Samples** | 36,3 | 30,1 | 17 | 36,3 | 29,5 | 19 | 36,3 | 29,4 | 19 |
|  | **High Samples** | 50,2 | 45,5 | 9 | 50,2 | 59,8 | 19 | 50,2 | 58,8 | 17 |
| **LC-1** | **Negative Samples** | <3 | <3 | 0 | <3 | <3 | 0 | <3 | <3 | 0 |
|  | **Low Samples** | 24,4 | 29,0 | 19 | 24,4 | 27,7 | 14 | 24,4 | 19,9 | 18 |
|  | **High Samples** | 48,5 | 49,6 | 2 | 48,5 | 52,4 | 8 | 48,5 | 54,3 | 12 |
| **SLA/LP** | **Negative Samples** | <3 | <3 | 0 | <3 | <3 | 0 | <3 | <3 | 0 |
|  | **Low Samples** | 32,2 | 26,3 | 18 | 32,2 | 29,7 | 8 | 32,2 | 28,2 | 12 |
|  | **High Samples** | 59,9 | 64,1 | 7 | 59,9 | 58,8 | 2 | 59,9 | 64,7 | 8 |

**Supplementary Table 3** Analytical analysis in presence of increasing concentration of triglyceride

|  |  | **Triglyceride** | | | | | | | | |
| --- | --- | --- | --- | --- | --- | --- | --- | --- | --- | --- |
| **Parameter** | **Sample** | **Level 1 (1500 mg/dL)** | | | **Level 2 (750 mg/dL)** | | | **Level 3 (250 mg/dL)** | | |
|  |  | **Control AU/mL** | **Spiked AU/mL** | **%diff** | **Control AU/mL** | **Spiked AU/mL** | **%diff** | **Control AU/mL** | **Spiked AU/mL** | **%diff** |
| **AMA** | **Negative Samples** | <5 | <5 | 0 | <5 | <5 | 0 | <5 | <5 | 0 |
|  | **Low Samples** | 25,9 | 24,4 | 6 | 25,9 | 30,1 | 16 | 25,9 | 30,1 | 16 |
|  | **High Samples** | 52,8 | 55,5 | 5 | 52,8 | 55,4 | 5 | 52,8 | 60,3 | 14 |
| **Sp100** | **Negative Samples** | <4 | <4 | 0 | <4 | <4 | 0 | <4 | <4 | 0 |
|  | **Low Samples** | 34,3 | 31,1 | 9 | 34,3 | 30,2 | 12 | 34,3 | 40,1 | 17 |
|  | **High Samples** | 66,2 | 60,2 | 9 | 66,2 | 64,1 | 3 | 66,2 | 63,9 | 3 |
| **gp210** | **Negative Samples** | <7 | <7 | 0 | <7 | <7 | 0 | <7 | <7 | 0 |
|  | **Low Samples** | 28,4 | 30,3 | 7 | 28,4 | 28,8 | 1 | 28,4 | 25,1 | 12 |
|  | **High Samples** | 59,6 | 69,5 | 17 | 59,6 | 66,3 | 11 | 59,6 | 55,4 | 7 |
| **LKM-1** | **Negative Samples** | <3 | <3 | 0 | <3 | <3 | 0 | <3 | <3 | 0 |
|  | **Low Samples** | 36,3 | 29,9 | 18 | 36,3 | 29,5 | 19 | 36,3 | 29,4 | 19 |
|  | **High Samples** | 50,2 | 56,9 | 13 | 50,2 | 59,1 | 18 | 50,2 | 54,4 | 8 |
| **LC-1** | **Negative Samples** | <3 | <3 | 0 | <3 | <3 | 0 | <3 | <3 | 0 |
|  | **Low Samples** | 24,4 | 28,8 | 18 | 24,4 | 27,7 | 14 | 24,4 | 19,9 | 18 |
|  | **High Samples** | 48,5 | 52,7 | 9 | 48,5 | 52,4 | 8 | 48,5 | 54,3 | 12 |
| **SLA/LP** | **Negative Samples** | <3 | <3 | 0 | <3 | <3 | 0 | <3 | <3 | 0 |
|  | **Low Samples** | 32,2 | 27,2 | 16 | 32,2 | 28,8 | 11 | 32,2 | 31,1 | 3 |
|  | **High Samples** | 59,9 | 54,4 | 9 | 59,9 | 55,0 | 8 | 59,9 | 64,1 | 7 |

**Supplementary Table 4** Analytical analysis in presence of increasing concentration of Rheumatoid factor

|  |  | **Rheumatoid Factor** | | | | | | | | |
| --- | --- | --- | --- | --- | --- | --- | --- | --- | --- | --- |
| **Parameter** | **Sample** | **Level 1 (220 UI/L)** | | | **Level 2 (110 UI/L)** | | | **Level 3 (44 UI/L)** | | |
|  |  | **Control AU/mL** | **Spiked AU/mL** | **%diff** | **Control AU/mL** | **Spiked AU/mL** | **%diff** | **Control AU/mL** | **Spiked AU/mL** | **%diff** |
| **AMA** | **Negative Samples** | <5 | <5 | 0 | <5 | <5 | 0 | <5 | <5 | 0 |
|  | **Low Samples** | 25,9 | 22,2 | 14 | 25,9 | 25,1 | 3 | 25,9 | 27,7 | 7 |
|  | **High Samples** | 52,8 | 48,2 | 9 | 52,8 | 50,1 | 5 | 52,8 | 55,8 | 6 |
| **Sp100** | **Negative Samples** | <4 | <4 | 0 | <4 | <4 | 0 | <4 | <4 | 0 |
|  | **Low Samples** | 34,3 | 29,2 | 15 | 34,3 | 33,3 | 3 | 34,3 | 37,7 | 10 |
|  | **High Samples** | 66,2 | 60,3 | 9 | 66,2 | 65,2 | 2 | 66,2 | 67,0 | 1 |
| **gp210** | **Negative Samples** | <7 | <7 | 0 | <7 | <7 | 0 | <7 | <7 | 0 |
|  | **Low Samples** | 28,4 | 25,5 | 10 | 28,4 | 31,1 | 10 | 28,4 | 29,6 | 4 |
|  | **High Samples** | 59,6 | 55,7 | 7 | 59,6 | 54,4 | 9 | 59,6 | 57,1 | 4 |
| **LKM-1** | **Negative Samples** | <3 | <3 | 0 | <3 | <3 | 0 | <3 | <3 | 0 |
|  | **Low Samples** | 36,3 | 30,2 | 17 | 36,3 | 34,4 | 5 | 36,3 | 33,0 | 9 |
|  | **High Samples** | 50,2 | 58,8 | 17 | 50,2 | 62,2 | 24 | 50,2 | 62,8 | 25 |
| **LC-1** | **Negative Samples** | <3 | <3 | 0 | <3 | <3 | 0 | <3 | <3 | 0 |
|  | **Low Samples** | 24,4 | 22,3 | 9 | 24,4 | 25,5 | 5 | 24,4 | 27,1 | 11 |
|  | **High Samples** | 48,5 | 45,5 | 6 | 48,5 | 44,7 | 8 | 48,5 | 49,5 | 2 |
| **SLA/LP** | **Negative Samples** | <3 | <3 | 0 | <3 | <3 | 0 | <3 | <3 | 0 |
|  | **Low Samples** | 32,2 | 31,5 | 2 | 32,2 | 30,3 | 6 | 32,2 | 26,9 | 16 |
|  | **High Samples** | 59,9 | 57,7 | 4 | 59,9 | 62,4 | 4 | 59,9 | 66,3 | 11 |

**Supplementary Table 5A** Cross-reactivity data for AMA, Sp100, and gp210

| **No** | **Sample code** | **Cross-reagent** | **Cross-reagent Characteristics** | **Parameter** | **AU/mL** | **Result** | **Expected result** | **Parameter** | **AU/mL** | **Result** | **Expected result** | **Parameter** | **AU/mL** | **Result** | **Expected result** |  |
| --- | --- | --- | --- | --- | --- | --- | --- | --- | --- | --- | --- | --- | --- | --- | --- | --- |
|  |  |  |  |  |  |  |  |  |  |  |  |  |  |  |  |  |
| **1** | **HCV 130** | **HEPATITIS-C** | IgG pos | AMA | <5 | N | <13,5 | Sp100 | <4 | N | <17,0 | gp210 | <7 | N | <18,0 |  |
| **2** | **HCV 131** | **HEPATITIS-C** | IgG pos | AMA | 13,1 | N | <13,5 | Sp100 | <4 | N | <17,0 | gp210 | <7 | N | <18,0 |  |
| **3** | **HCV 132** | **HEPATITIS-C** | IgG pos | AMA | <5 | N | <13,5 | Sp100 | <4 | N | <17,0 | gp210 | <7 | N | <18,0 |  |
| **4** | **HCV 133** | **HEPATITIS-C** | IgG pos | AMA | <5 | N | <13,5 | Sp100 | <4 | N | <17,0 | gp210 | <7 | N | <18,0 |  |
| **5** | **HCV 134** | **HEPATITIS-C** | IgG pos | AMA | <5 | N | <13,5 | Sp100 | <4 | N | <17,0 | gp210 | <7 | N | <18,0 |  |
| **6** | **HCV 135** | **HEPATITIS-C** | IgG pos | AMA | <5 | N | <13,5 | Sp100 | <4 | N | <17,0 | gp210 | <7 | N | <18,0 |  |
| **7** | **HCV 136** | **HEPATITIS-C** | IgG pos | AMA | <5 | N | <13,5 | Sp100 | <4 | N | <17,0 | gp210 | <7 | N | <18,0 |  |
| **8** | **E-22614** | **HEPATITIS-C** | IgG pos | AMA | <5 | N | <13,5 | Sp100 | <4 | N | <17,0 | gp210 | <7 | N | <18,0 |  |
| **9** | **E-22615** | **HEPATITIS-C** | IgG pos | AMA | <5 | N | <13,5 | Sp100 | <4 | N | <17,0 | gp210 | <7 | N | <18,0 |  |
| **10** | **E-22616** | **HEPATITIS-C** | IgG pos | AMA | <5 | N | <13,5 | Sp100 | <4 | N | <17,0 | gp210 | <7 | N | <18,0 |  |
| **11** | **E-22617** | **HEPATITIS-C** | IgG pos | AMA | <5 | N | <13,5 | Sp100 | <4 | N | <17,0 | gp210 | <7 | N | <18,0 |  |
| **12** | **E-22618** | **HEPATITIS-C** | IgG pos | AMA | <5 | N | <13,5 | Sp100 | <4 | N | <17,0 | gp210 | <7 | N | <18,0 |  |
| **13** | **E-22619** | **HEPATITIS-C** | IgG pos | AMA | <5 | N | <13,5 | Sp100 | <4 | N | <17,0 | gp210 | <7 | N | <18,0 |  |
| **14** | **E-22620** | **HEPATITIS-C** | IgG pos | AMA | <5 | N | <13,5 | Sp100 | <4 | N | <17,0 | gp210 | <7 | N | <18,0 |  |
| **15** | **E-22621** | **HEPATITIS-C** | IgG pos | AMA | <5 | N | <13,5 | Sp100 | <4 | N | <17,0 | gp210 | <7 | N | <18,0 |  |
| **16** | **E-22622** | **HEPATITIS-C** | IgG pos | AMA | <5 | N | <13,5 | Sp100 | <4 | N | <17,0 | gp210 | <7 | N | <18,0 |  |
| **17** | **E-22623** | **HEPATITIS-C** | IgG pos | AMA | <5 | N | <13,5 | Sp100 | <4 | N | <17,0 | gp210 | <7 | N | <18,0 |  |
| **18** | **E-22624** | **HEPATITIS-C** | IgG pos | AMA | <5 | N | <13,5 | Sp100 | <4 | N | <17,0 | gp210 | <7 | N | <18,0 |  |
| **19** | **E-22625** | **HEPATITIS-C** | IgG pos | AMA | <5 | N | <13,5 | Sp100 | <4 | N | <17,0 | gp210 | <7 | N | <18,0 |  |
| **20** | **E-22626** | **HEPATITIS-C** | IgG pos | AMA | <5 | N | <13,5 | Sp100 | <4 | N | <17,0 | gp210 | <7 | N | <18,0 |  |
| **21** | **E-22627** | **HEPATITIS-C** | IgG pos | AMA | <5 | N | <13,5 | Sp100 | <4 | N | <17,0 | gp210 | <7 | N | <18,0 |  |
| **22** | **E-22628** | **HEPATITIS-C** | IgG pos | AMA | <5 | N | <13,5 | Sp100 | <4 | N | <17,0 | gp210 | <7 | N | <18,0 |  |
| **23** | **E-21547** | **HEPATITIS-C** | IgG pos | AMA | <5 | N | <13,5 | Sp100 | <4 | N | <17,0 | gp210 | <7 | N | <18,0 |  |
| **24** | **E-21548** | **HEPATITIS-C** | IgG pos | AMA | <5 | N | <13,5 | Sp100 | <4 | N | <17,0 | gp210 | <7 | N | <18,0 |  |
| **25** | **E-21549** | **HEPATITIS-C** | IgG pos | AMA | <5 | N | <13,5 | Sp100 | <4 | N | <17,0 | gp210 | <7 | N | <18,0 |  |
| **26** | **E-21550** | **HEPATITIS-C** | IgG pos | AMA | <5 | N | <13,5 | Sp100 | <4 | N | <17,0 | gp210 | <7 | N | <18,0 |  |
| **27** | **E-21528** | **HEPATITIS-C** | IgG pos | AMA | <5 | N | <13,5 | Sp100 | <4 | N | <17,0 | gp210 | <7 | N | <18,0 |  |
| **28** | **E-21529** | **HEPATITIS-C** | IgG pos | AMA | <5 | N | <13,5 | Sp100 | <4 | N | <17,0 | gp210 | <7 | N | <18,0 |  |
| **29** | **E-21530** | **HEPATITIS-C** | IgG pos | AMA | <5 | N | <13,5 | Sp100 | <4 | N | <17,0 | gp210 | <7 | N | <18,0 |  |
| **30** | **E-21604** | **HEPATITIS-C** | IgG pos | AMA | <5 | N | <13,5 | Sp100 | <4 | N | <17,0 | gp210 | <7 | N | <18,0 |  |
| **31** | **ANCA 32** | **PR3 Pos** | IgG pos | AMA | <5 | N | <13,5 | Sp100 | <4 | N | <17,0 | gp210 | <7 | N | <18,0 |  |
| **32** | **ANCA 33** | **PR3 Pos** | IgG pos | AMA | <5 | N | <13,5 | Sp100 | <4 | N | <17,0 | gp210 | <7 | N | <18,0 |  |
| **33** | **ANCA 34** | **PR3 Pos** | IgG pos | AMA | <5 | N | <13,5 | Sp100 | <4 | N | <17,0 | gp210 | <7 | N | <18,0 |  |
| **34** | **ANCA 37** | **PR3 Pos** | IgG pos | AMA | <5 | N | <13,5 | Sp100 | <4 | N | <17,0 | gp210 | <7 | N | <18,0 |  |
| **35** | **ANCA 38** | **PR3 Pos** | IgG pos | AMA | <5 | N | <13,5 | Sp100 | <4 | N | <17,0 | gp210 | <7 | N | <18,0 |  |
| **36** | **ANCA 39** | **PR3 Pos** | IgG pos | AMA | <5 | N | <13,5 | Sp100 | <4 | N | <17,0 | gp210 | <7 | N | <18,0 |  |
| **37** | **ANCA 41** | **PR3 Pos** | IgG pos | AMA | <5 | N | <13,5 | Sp100 | <4 | N | <17,0 | gp210 | <7 | N | <18,0 |  |
| **38** | **ANCA 19** | **PR3 Pos** | IgG pos | AMA | <5 | N | <13,5 | Sp100 | <4 | N | <17,0 | gp210 | <7 | N | <18,0 |  |
| **39** | **ANCA 24** | **PR3 Pos** | IgG pos | AMA | <5 | N | <13,5 | Sp100 | <4 | N | <17,0 | gp210 | <7 | N | <18,0 |  |
| **40** | **ANCA 25** | **PR3 Pos** | IgG pos | AMA | <5 | N | <13,5 | Sp100 | <4 | N | <17,0 | gp210 | <7 | N | <18,0 |  |
| **41** | **ANCA 28** | **PR3 Pos** | IgG pos | AMA | <5 | N | <13,5 | Sp100 | <4 | N | <17,0 | gp210 | <7 | N | <18,0 |  |
| **42** | **ANCA 29** | **PR3 Pos** | IgG pos | AMA | <5 | N | <13,5 | Sp100 | <4 | N | <17,0 | gp210 | <7 | N | <18,0 |  |
| **43** | **ANCA 30** | **PR3 Pos** | IgG pos | AMA | <5 | N | <13,5 | Sp100 | <4 | N | <17,0 | gp210 | <7 | N | <18,0 |  |
| **44** | **ANCA 31** | **PR3 Pos** | IgG pos | AMA | <5 | N | <13,5 | Sp100 | <4 | N | <17,0 | gp210 | <7 | N | <18,0 |  |
| **45** | **ANCA 107** | **MPO Pos** | IgG pos | AMA | <5 | N | <13,5 | Sp100 | <4 | N | <17,0 | gp210 | <7 | N | <18,0 |  |
| **46** | **ANCA 108** | **MPO Pos** | IgG pos | AMA | <5 | N | <13,5 | Sp100 | <4 | N | <17,0 | gp210 | <7 | N | <18,0 |  |
| **47** | **ANCA 109** | **MPO Pos** | IgG pos | AMA | <5 | N | <13,5 | Sp100 | <4 | N | <17,0 | gp210 | <7 | N | <18,0 |  |
| **48** | **ANCA 97** | **MPO Pos** | IgG pos | AMA | <5 | N | <13,5 | Sp100 | <4 | N | <17,0 | gp210 | <7 | N | <18,0 |  |
| **49** | **ANCA 3** | **MPO Pos** | IgG pos | AMA | <5 | N | <13,5 | Sp100 | <4 | N | <17,0 | gp210 | <7 | N | <18,0 |  |
| **50** | **ANCA 15** | **MPO Pos** | IgG pos | AMA | <5 | N | <13,5 | Sp100 | <4 | N | <17,0 | gp210 | <7 | N | <18,0 |  |
| **51** | **ANCA 26** | **MPO Pos** | IgG pos | AMA | <5 | N | <13,5 | Sp100 | <4 | N | <17,0 | gp210 | <7 | N | <18,0 |  |
| **52** | **ANA 1** | **SS-A Pos** | IgG pos | AMA | <5 | N | <13,5 | Sp100 | <4 | N | <17,0 | gp210 | <7 | N | <18,0 |  |
| **53** | **ANA2** | **SS-A Pos** | IgG pos | AMA | <5 | N | <13,5 | Sp100 | <4 | N | <17,0 | gp210 | <7 | N | <18,0 |  |
| **54** | **ANA 3** | **SS-A Pos** | IgG pos | AMA | <5 | N | <13,5 | Sp100 | <4 | N | <17,0 | gp210 | <7 | N | <18,0 |  |
| **55** | **ANA 4** | **SS-B Pos** | IgG pos | AMA | <5 | N | <13,5 | Sp100 | <4 | N | <17,0 | gp210 | <7 | N | <18,0 |  |
| **56** | **ANA 5** | **SS-B Pos** | IgG pos | AMA | <5 | N | <13,5 | Sp100 | <4 | N | <17,0 | gp210 | <7 | N | <18,0 |  |
| **57** | **ANA 6** | **SS-B Pos** | IgG pos | AMA | <5 | N | <13,5 | Sp100 | <4 | N | <17,0 | gp210 | <7 | N | <18,0 |  |
| **58** | **ANA 7** | **CENP-B Pos** | IgG pos | AMA | <5 | N | <13,5 | Sp100 | <4 | N | <17,0 | gp210 | <7 | N | <18,0 |  |
| **59** | **ANA 8** | **CENP-B Pos** | IgG pos | AMA | <5 | N | <13,5 | Sp100 | <4 | N | <17,0 | gp210 | <7 | N | <18,0 |  |
| **60** | **ANA 9** | **CENP-B Pos** | IgG pos | AMA | <5 | N | <13,5 | Sp100 | <4 | N | <17,0 | gp210 | <7 | N | <18,0 |  |
| **61** | **ANA 10** | **CENP-B Pos** | IgG pos | AMA | <5 | N | <13,5 | Sp100 | <4 | N | <17,0 | gp210 | <7 | N | <18,0 |  |
| **62** | **ANA 11** | **CENP-B Pos** | IgG pos | AMA | <5 | N | <13,5 | Sp100 | <4 | N | <17,0 | gp210 | <7 | N | <18,0 |  |
| **63** | **ANA 12** | **dsDNA-G Pos** | IgG pos | AMA | <5 | N | <13,5 | Sp100 | <4 | N | <17,0 | gp210 | <7 | N | <18,0 |  |
| **64** | **ANA 13** | **dsDNA-G Pos** | IgG pos | AMA | <5 | N | <13,5 | Sp100 | <4 | N | <17,0 | gp210 | <7 | N | <18,0 |  |
| **65** | **ANA 14** | **SCL-70 Pos** | IgG pos | AMA | <5 | N | <13,5 | Sp100 | <4 | N | <17,0 | gp210 | <7 | N | <18,0 |  |
| **66** | **ANA 15** | **SCL-70 Pos** | IgG pos | AMA | <5 | N | <13,5 | Sp100 | <4 | N | <17,0 | gp210 | <7 | N | <18,0 |  |
| **67** | **ANA 16** | **SCL-70 Pos** | IgG pos | AMA | <5 | N | <13,5 | Sp100 | <4 | N | <17,0 | gp210 | <7 | N | <18,0 |  |
| **68** | **ANA 17** | **JO-1 Pos** | IgG pos | AMA | <5 | N | <13,5 | Sp100 | <4 | N | <17,0 | gp210 | <7 | N | <18,0 |  |
| **69** | **ANA 18** | **JO-1 Pos** | IgG pos | AMA | <5 | N | <13,5 | Sp100 | <4 | N | <17,0 | gp210 | <7 | N | <18,0 |  |
| **70** | **ANA 19** | **snRNP Pos** | IgG pos | AMA | <5 | N | <13,5 | Sp100 | <4 | N | <17,0 | gp210 | <7 | N | <18,0 |  |
| **71** | **ANA 20** | **snRNP Pos** | IgG pos | AMA | <5 | N | <13,5 | Sp100 | <4 | N | <17,0 | gp210 | <7 | N | <18,0 |  |
| **72** | **ANA 21** | **snRNP Pos** | IgG pos | AMA | <5 | N | <13,5 | Sp100 | <4 | N | <17,0 | gp210 | <7 | N | <18,0 |  |
| **73** | **AIH/CBP 157** | **SMA Pos** | IgG pos | AMA | <5 | N | <13,5 | Sp100 | <4 | N | <17,0 | gp210 | <7 | N | <18,0 |  |

**Supplementary Table 5B** Cross-reactivity data for LKM-1, LC-1, and SLA/LP

| **No** | **Sample code** | **Cross-reagent** | **Cross-reagent Characteristics** | **Parameter** | **AU/mL** | **Result** | **Expected result** | **Parameter** | **AU/mL** | **Result** | **Expected result** | **Parameter** | **AU/mL** | **Result** | **Expected result** |  |
| --- | --- | --- | --- | --- | --- | --- | --- | --- | --- | --- | --- | --- | --- | --- | --- | --- |
|  |  |  |  |  |  |  |  |  |  |  |  |  |  |  |  |  |
| **1** | **HCV 130** | **HEPATITIS-C** | IgG pos | LKM-1 | <3 | N | <17,0 | LC-1 | <3 | N | <16,0 | SLA/LP | <3 | N | <20,0 |  |
| **2** | **HCV 131** | **HEPATITIS-C** | IgG pos | LKM-1 | <3 | N | <17,0 | LC-1 | 7,65 | N | <16,0 | SLA/LP | <3 | N | <20,0 |  |
| **3** | **HCV 132** | **HEPATITIS-C** | IgG pos | LKM-1 | <3 | N | <17,0 | LC-1 | <3 | N | <16,0 | SLA/LP | <3 | N | <20,0 |  |
| **4** | **HCV 133** | **HEPATITIS-C** | IgG pos | LKM-1 | <3 | N | <17,0 | LC-1 | <3 | N | <16,0 | SLA/LP | <3 | N | <20,0 |  |
| **5** | **HCV 134** | **HEPATITIS-C** | IgG pos | LKM-1 | <3 | N | <17,0 | LC-1 | 6,18 | N | <16,0 | SLA/LP | <3 | N | <20,0 |  |
| **6** | **HCV 135** | **HEPATITIS-C** | IgG pos | LKM-1 | <3 | N | <17,0 | LC-1 | <3 | N | <16,0 | SLA/LP | <3 | N | <20,0 |  |
| **7** | **HCV 136** | **HEPATITIS-C** | IgG pos | LKM-1 | <3 | N | <17,0 | LC-1 | <3 | N | <16,0 | SLA/LP | <3 | N | <20,0 |  |
| **8** | **E-22614** | **HEPATITIS-C** | IgG pos | LKM-1 | <3 | N | <17,0 | LC-1 | <3 | N | <16,0 | SLA/LP | <3 | N | <20,0 |  |
| **9** | **E-22615** | **HEPATITIS-C** | IgG pos | LKM-1 | <3 | N | <17,0 | LC-1 | <3 | N | <16,0 | SLA/LP | <3 | N | <20,0 |  |
| **10** | **E-22616** | **HEPATITIS-C** | IgG pos | LKM-1 | <3 | N | <17,0 | LC-1 | <3 | N | <16,0 | SLA/LP | <3 | N | <20,0 |  |
| **11** | **E-22617** | **HEPATITIS-C** | IgG pos | LKM-1 | <3 | N | <17,0 | LC-1 | <3 | N | <16,0 | SLA/LP | <3 | N | <20,0 |  |
| **12** | **E-22618** | **HEPATITIS-C** | IgG pos | LKM-1 | <3 | N | <17,0 | LC-1 | <3 | N | <16,0 | SLA/LP | <3 | N | <20,0 |  |
| **13** | **E-22619** | **HEPATITIS-C** | IgG pos | LKM-1 | <3 | N | <17,0 | LC-1 | <3 | N | <16,0 | SLA/LP | <3 | N | <20,0 |  |
| **14** | **E-22620** | **HEPATITIS-C** | IgG pos | LKM-1 | <3 | N | <17,0 | LC-1 | <3 | N | <16,0 | SLA/LP | <3 | N | <20,0 |  |
| **15** | **E-22621** | **HEPATITIS-C** | IgG pos | LKM-1 | <3 | N | <17,0 | LC-1 | <3 | N | <16,0 | SLA/LP | <3 | N | <20,0 |  |
| **16** | **E-22622** | **HEPATITIS-C** | IgG pos | LKM-1 | <3 | N | <17,0 | LC-1 | <3 | N | <16,0 | SLA/LP | <3 | N | <20,0 |  |
| **17** | **E-22623** | **HEPATITIS-C** | IgG pos | LKM-1 | <3 | N | <17,0 | LC-1 | <3 | N | <16,0 | SLA/LP | <3 | N | <20,0 |  |
| **18** | **E-22624** | **HEPATITIS-C** | IgG pos | LKM-1 | <3 | N | <17,0 | LC-1 | <3 | N | <16,0 | SLA/LP | <3 | N | <20,0 |  |
| **19** | **E-22625** | **HEPATITIS-C** | IgG pos | LKM-1 | <3 | N | <17,0 | LC-1 | <3 | N | <16,0 | SLA/LP | <3 | N | <20,0 |  |
| **20** | **E-22626** | **HEPATITIS-C** | IgG pos | LKM-1 | <3 | N | <17,0 | LC-1 | <3 | N | <16,0 | SLA/LP | <3 | N | <20,0 |  |
| **21** | **E-22627** | **HEPATITIS-C** | IgG pos | LKM-1 | <3 | N | <17,0 | LC-1 | <3 | N | <16,0 | SLA/LP | <3 | N | <20,0 |  |
| **22** | **E-22628** | **HEPATITIS-C** | IgG pos | LKM-1 | <3 | N | <17,0 | LC-1 | <3 | N | <16,0 | SLA/LP | <3 | N | <20,0 |  |
| **23** | **E-21547** | **HEPATITIS-C** | IgG pos | LKM-1 | <3 | N | <17,0 | LC-1 | <3 | N | <16,0 | SLA/LP | <3 | N | <20,0 |  |
| **24** | **E-21548** | **HEPATITIS-C** | IgG pos | LKM-1 | <3 | N | <17,0 | LC-1 | <3 | N | <16,0 | SLA/LP | <3 | N | <20,0 |  |
| **25** | **E-21549** | **HEPATITIS-C** | IgG pos | LKM-1 | <3 | N | <17,0 | LC-1 | <3 | N | <16,0 | SLA/LP | <3 | N | <20,0 |  |
| **26** | **E-21550** | **HEPATITIS-C** | IgG pos | LKM-1 | <3 | N | <17,0 | LC-1 | <3 | N | <16,0 | SLA/LP | <3 | N | <20,0 |  |
| **27** | **E-21528** | **HEPATITIS-C** | IgG pos | LKM-1 | <3 | N | <17,0 | LC-1 | <3 | N | <16,0 | SLA/LP | <3 | N | <20,0 |  |
| **28** | **E-21529** | **HEPATITIS-C** | IgG pos | LKM-1 | <3 | N | <17,0 | LC-1 | <3 | N | <16,0 | SLA/LP | <3 | N | <20,0 |  |
| **29** | **E-21530** | **HEPATITIS-C** | IgG pos | LKM-1 | <3 | N | <17,0 | LC-1 | <3 | N | <16,0 | SLA/LP | <3 | N | <20,0 |  |
| **30** | **E-21604** | **HEPATITIS-C** | IgG pos | LKM-1 | <3 | N | <17,0 | LC-1 | <3 | N | <16,0 | SLA/LP | <3 | N | <20,0 |  |
| **31** | **ANCA 32** | **PR3 Pos** | IgG pos | LKM-1 | <3 | N | <17,0 | LC-1 | <3 | N | <16,0 | SLA/LP | <3 | N | <20,0 |  |
| **32** | **ANCA 33** | **PR3 Pos** | IgG pos | LKM-1 | <3 | N | <17,0 | LC-1 | <3 | N | <16,0 | SLA/LP | <3 | N | <20,0 |  |
| **33** | **ANCA 34** | **PR3 Pos** | IgG pos | LKM-1 | <3 | N | <17,0 | LC-1 | <3 | N | <16,0 | SLA/LP | <3 | N | <20,0 |  |
| **34** | **ANCA 37** | **PR3 Pos** | IgG pos | LKM-1 | <3 | N | <17,0 | LC-1 | <3 | N | <16,0 | SLA/LP | <3 | N | <20,0 |  |
| **35** | **ANCA 38** | **PR3 Pos** | IgG pos | LKM-1 | <3 | N | <17,0 | LC-1 | <3 | N | <16,0 | SLA/LP | <3 | N | <20,0 |  |
| **36** | **ANCA 39** | **PR3 Pos** | IgG pos | LKM-1 | <3 | N | <17,0 | LC-1 | <3 | N | <16,0 | SLA/LP | <3 | N | <20,0 |  |
| **37** | **ANCA 41** | **PR3 Pos** | IgG pos | LKM-1 | <3 | N | <17,0 | LC-1 | <3 | N | <16,0 | SLA/LP | <3 | N | <20,0 |  |
| **38** | **ANCA 19** | **PR3 Pos** | IgG pos | LKM-1 | <3 | N | <17,0 | LC-1 | <3 | N | <16,0 | SLA/LP | <3 | N | <20,0 |  |
| **39** | **ANCA 24** | **PR3 Pos** | IgG pos | LKM-1 | <3 | N | <17,0 | LC-1 | <3 | N | <16,0 | SLA/LP | <3 | N | <20,0 |  |
| **40** | **ANCA 25** | **PR3 Pos** | IgG pos | LKM-1 | <3 | N | <17,0 | LC-1 | <3 | N | <16,0 | SLA/LP | <3 | N | <20,0 |  |
| **41** | **ANCA 28** | **PR3 Pos** | IgG pos | LKM-1 | <3 | N | <17,0 | LC-1 | <3 | N | <16,0 | SLA/LP | <3 | N | <20,0 |  |
| **42** | **ANCA 29** | **PR3 Pos** | IgG pos | LKM-1 | <3 | N | <17,0 | LC-1 | <3 | N | <16,0 | SLA/LP | <3 | N | <20,0 |  |
| **43** | **ANCA 30** | **PR3 Pos** | IgG pos | LKM-1 | <3 | N | <17,0 | LC-1 | <3 | N | <16,0 | SLA/LP | <3 | N | <20,0 |  |
| **44** | **ANCA 31** | **PR3 Pos** | IgG pos | LKM-1 | <3 | N | <17,0 | LC-1 | <3 | N | <16,0 | SLA/LP | <3 | N | <20,0 |  |
| **45** | **ANCA 107** | **MPO Pos** | IgG pos | LKM-1 | <3 | N | <17,0 | LC-1 | <3 | N | <16,0 | SLA/LP | <3 | N | <20,0 |  |
| **46** | **ANCA 108** | **MPO Pos** | IgG pos | LKM-1 | <3 | N | <17,0 | LC-1 | <3 | N | <16,0 | SLA/LP | <3 | N | <20,0 |  |
| **47** | **ANCA 109** | **MPO Pos** | IgG pos | LKM-1 | <3 | N | <17,0 | LC-1 | <3 | N | <16,0 | SLA/LP | <3 | N | <20,0 |  |
| **48** | **ANCA 97** | **MPO Pos** | IgG pos | LKM-1 | <3 | N | <17,0 | LC-1 | <3 | N | <16,0 | SLA/LP | <3 | N | <20,0 |  |
| **49** | **ANCA 3** | **MPO Pos** | IgG pos | LKM-1 | <3 | N | <17,0 | LC-1 | <3 | N | <16,0 | SLA/LP | <3 | N | <20,0 |  |
| **50** | **ANCA 15** | **MPO Pos** | IgG pos | LKM-1 | <3 | N | <17,0 | LC-1 | <3 | N | <16,0 | SLA/LP | <3 | N | <20,0 |  |
| **51** | **ANCA 26** | **MPO Pos** | IgG pos | LKM-1 | <3 | N | <17,0 | LC-1 | <3 | N | <16,0 | SLA/LP | <3 | N | <20,0 |  |
| **52** | **ANA 1** | **SS-A Pos** | IgG pos | LKM-1 | <3 | N | <17,0 | LC-1 | <3 | N | <16,0 | SLA/LP | <3 | N | <20,0 |  |
| **53** | **ANA2** | **SS-A Pos** | IgG pos | LKM-1 | <3 | N | <17,0 | LC-1 | <3 | N | <16,0 | SLA/LP | <3 | N | <20,0 |  |
| **54** | **ANA 3** | **SS-A Pos** | IgG pos | LKM-1 | <3 | N | <17,0 | LC-1 | <3 | N | <16,0 | SLA/LP | <3 | N | <20,0 |  |
| **55** | **ANA 4** | **SS-B Pos** | IgG pos | LKM-1 | <3 | N | <17,0 | LC-1 | <3 | N | <16,0 | SLA/LP | <3 | N | <20,0 |  |
| **56** | **ANA 5** | **SS-B Pos** | IgG pos | LKM-1 | <3 | N | <17,0 | LC-1 | <3 | N | <16,0 | SLA/LP | <3 | N | <20,0 |  |
| **57** | **ANA 6** | **SS-B Pos** | IgG pos | LKM-1 | <3 | N | <17,0 | LC-1 | <3 | N | <16,0 | SLA/LP | <3 | N | <20,0 |  |
| **58** | **ANA 7** | **CENP-B Pos** | IgG pos | LKM-1 | <3 | N | <17,0 | LC-1 | <3 | N | <16,0 | SLA/LP | <3 | N | <20,0 |  |
| **59** | **ANA 8** | **CENP-B Pos** | IgG pos | LKM-1 | <3 | N | <17,0 | LC-1 | <3 | N | <16,0 | SLA/LP | <3 | N | <20,0 |  |
| **60** | **ANA 9** | **CENP-B Pos** | IgG pos | LKM-1 | <3 | N | <17,0 | LC-1 | <3 | N | <16,0 | SLA/LP | <3 | N | <20,0 |  |
| **61** | **ANA 10** | **CENP-B Pos** | IgG pos | LKM-1 | <3 | N | <17,0 | LC-1 | <3 | N | <16,0 | SLA/LP | <3 | N | <20,0 |  |
| **62** | **ANA 11** | **CENP-B Pos** | IgG pos | LKM-1 | <3 | N | <17,0 | LC-1 | <3 | N | <16,0 | SLA/LP | <3 | N | <20,0 |  |
| **63** | **ANA 12** | **dsDNA-G Pos** | IgG pos | LKM-1 | <3 | N | <17,0 | LC-1 | <3 | N | <16,0 | SLA/LP | <3 | N | <20,0 |  |
| **64** | **ANA 13** | **dsDNA-G Pos** | IgG pos | LKM-1 | <3 | N | <17,0 | LC-1 | <3 | N | <16,0 | SLA/LP | <3 | N | <20,0 |  |
| **65** | **ANA 14** | **SCL-70 Pos** | IgG pos | LKM-1 | <3 | N | <17,0 | LC-1 | <3 | N | <16,0 | SLA/LP | <3 | N | <20,0 |  |
| **66** | **ANA 15** | **SCL-70 Pos** | IgG pos | LKM-1 | <3 | N | <17,0 | LC-1 | <3 | N | <16,0 | SLA/LP | <3 | N | <20,0 |  |
| **67** | **ANA 16** | **SCL-70 Pos** | IgG pos | LKM-1 | <3 | N | <17,0 | LC-1 | <3 | N | <16,0 | SLA/LP | <3 | N | <20,0 |  |
| **68** | **ANA 17** | **JO-1 Pos** | IgG pos | LKM-1 | <3 | N | <17,0 | LC-1 | <3 | N | <16,0 | SLA/LP | <3 | N | <20,0 |  |
| **69** | **ANA 18** | **JO-1 Pos** | IgG pos | LKM-1 | <3 | N | <17,0 | LC-1 | <3 | N | <16,0 | SLA/LP | <3 | N | <20,0 |  |
| **70** | **ANA 19** | **snRNP Pos** | IgG pos | LKM-1 | <3 | N | <17,0 | LC-1 | <3 | N | <16,0 | SLA/LP | <3 | N | <20,0 |  |
| **71** | **ANA 20** | **snRNP Pos** | IgG pos | LKM-1 | <3 | N | <17,0 | LC-1 | <3 | N | <16,0 | SLA/LP | <3 | N | <20,0 |  |
| **72** | **ANA 21** | **snRNP Pos** | IgG pos | LKM-1 | <3 | N | <17,0 | LC-1 | <3 | N | <16,0 | SLA/LP | <3 | N | <20,0 |  |
| **73** | **AIH/CBP 157** | **SMA Pos** | IgG pos | LKM-1 | <3 | N | <17,0 | LC-1 | <3 | N | <16,0 | SLA/LP | <3 | N | <20,0 |  |
